# Testosterone is not associated with traits of optimism or pessimism: Observational evidence from the prospective DETECT study

**DOI:** 10.1371/journal.pone.0207870

**Published:** 2018-11-29

**Authors:** Hanna Kische, Jürgen Hoyer, Lars Pieper, John Venz, Jens Klotsche, Winfried März, Uwe Koch-Gromus, David Pittrow, Hendrik Lehnert, Sigmund Silber, Günter K. Stalla, Andreas M. Zeiher, Hans-Ulrich Wittchen, Robin Haring

**Affiliations:** 1 Behavioral Epidemiology, Institute of Clinical Psychology and Psychotherapy, Technical University of Dresden, Dresden, Germany; 2 Institute of Clinical Psychology and Psychotherapy, Technical University of Dresden, Dresden, Germany; 3 German Rheumatism Research Centre Berlin, Berlin, Germany; 4 Medical Clinic V, Medical Faculty Mannheim at Heidelberg University, Heidelberg, Germany; 5 Department of Medical Psychology, University Medical Center Eppendorf, Hamburg, Germany; 6 Institute of Clinical Pharmacology, Medical Faculty, Technical University of Dresden, Dresden, Germany; 7 Department of Medicine I, University of Schleswig-Holstein, Lübeck, Germany; 8 Cardiology Practice and Hospital, Munich, Germany; 9 Max Planck Institute of Psychiatry, Munich, Germany; 10 Department of Medicine III Cardiology, Goethe University Frankfurt, Frankfurt, Germany; 11 Institute of Clinical Psychology and Psychotherapy, Technical University of Dresden, Dresden, Germany; 12 European University of Applied Sciences, Faculty of Applied Public Health, Rostock, Germany; 13 School of Public Health and Preventive Medicine, Monash University, Melbourne, Australia; Western Sydney University, AUSTRALIA

## Abstract

**Background:**

Previous experimental research on testosterone (T) and psychological traits is inconclusive. Thus, we performed the first large-scale observational study of the association between T and dispositional optimism / pessimism.

**Methods:**

We used prospective data from 6,493 primary-care patients (3,840 women) of the DETECT study (Diabetes Cardiovascular Risk-Evaluation: Targets and Essential Data for Commitment of Treatment), including repeated immunoassay-based measurement of serum T and optimism / pessimism assessed by the revised Life-Orientation Test (LOT-R). Cross-sectional and longitudinal associations of baseline T and one-year change in T with optimism and pessimism were investigated using age- and multivariable-adjusted regression models.

**Results:**

Cross-sectional analyses showed no association of T with optimism or pessimism in both sexes. Longitudinal analyses also showed no association of baseline T with optimism or pessimism at four-year follow-up. Multivariable analyses of total LOT-R score yielded similarly non-significant results (β-coefficient per unit change in T for men: -0.01 (95% CI: -0.24–0.22), women: 0.08 (-0.03–0.20)). Furthermore, change in T was not related to optimism or pessimism at four-year follow-up.

**Conclusions:**

The present observational study of a large-scale prospective sample showed no association of T with optimism or pessimism. Integrating further experimental and interventional evidence from alternative methodological approaches would strengthen this conclusion and establish stronger evidence about the potential hormonal basis of psychological traits.

## Introduction

From its first use in the treatment of depression in 1948 [1], testosterone (T) has a long clinical history in the application for psychological disorders. Between 1950 and 2016, a recent systematic review identified 45 randomized controlled trials (RCT) investigating the effect of T treatment on personality, well-being or mood, but without any consistent effect on psychological outcomes [2].

The empirical evidence for a link between T and psychological traits, such as optimism and pessimism, is only weak. Initial support for a potential hormonal basis of psychological traits relies on findings from two different strands of literature. First, research on prenatal T provides tentative evidence that traits are—at least partially—biologically determined [3]. Secondly, experimental research in economic decision-making suggests a potential link of T with risk-taking, overconfidence and optimism [4]. However, the exact association between T and psychological traits is still elusive [5]. Therefore, we investigated the association of T with optimism and pessimism using cross-sectional and longitudinal data from a large patient-based sample of men and women.

## Methods

### Study population

The Diabetes Cardiovascular Risk-Evaluation: Targets and Essential Data for Commitment of Treatment (DETECT) study is a prospective primary-care study in Germany. Details of the study design, recruitment, and procedures were previously published [6]. In brief, of 55,518 eligible patients at baseline, a random subsample of 7,519 patients was recruited in 851 primary-care settings. Of these, 6,826 patients (2,782 men and 4,044 women) participated in one-year and/or four-year follow-up assessments between September and December 2004 and 2007, respectively. The follow-up response rate was 90.8%. All patients gave written informed consent and the study was approved by the ethics committee of the Technical University of Dresden. We excluded patients due to missing T data (N = 896), baseline age above 86 years (N = 30), and use of anti-androgens (N = 100). None of the women were pregnant. The final study comprised of 2,653 men and 3,840 women.

### Measurements

Socio-demographics and medical history were assessed by primary-care physicians using standardized interviews and medical records. Blood pressure, height, weight, and waist circumference were measured according to standardized instructions. For smoking, alcohol consumption, and physical activity we used the following categorized variables: Smoking was categorized into current smokers and non-smokers. Alcohol consumption was categorized in self-reported sobriety, infrequent, occasional or daily alcohol consumption. Participants who participated in physical activity during summer or winter for at least two hour a week were classified as being physically active.

The revised version of the 10 item Life Orientation Test (LOT-R) was used to define optimism as a sub-score including items 1, 4, and 10 and pessimism including items 3, 7, and 9, as previously described [7]. Repeated measurements of baseline and one-year follow-up T concentrations based on blood samples taken between 8.00 and 10.00 a.m. and were performed using an electrochemiluminescence immunoassay (Modular analytics, Roche Diagnostics, Mannheim, Germany) with an intra- and interassay coefficient of variation of 2.7% and 5.6%, respectively. Full laboratory procedures were published previously [8].

### Statistical analyses

First, cross-sectional and longitudinal associations of continuous T with optimism, pessimism, and total LOT-R score were analyzed using age- and multivariable-adjusted linear regression models, with effects reported as β-coefficients and their 95% confidence interval (CI). Second, T change was defined as absolute difference between baseline and one-year follow-up T, with associations between T change and LOT-R investigated by linear regression models. Normality of residuals was tested using QQ plots and normality of outcome variables was inspected visually. Multivariable models were adjusted for age, waist-circumference, smoking habits, physical activity, alcohol consumption, and blood pressure. To address potential attrition bias, we included inverse probability weights into longitudinal multivariable analyses. All analyses were performed with robust standard errors and p-values < 0.05 as threshold for statistical significance. Statistical analyses were performed with Stata 15.0 (Stata Corp., College Station, TX, USA).

## Results

**Fig 1:**
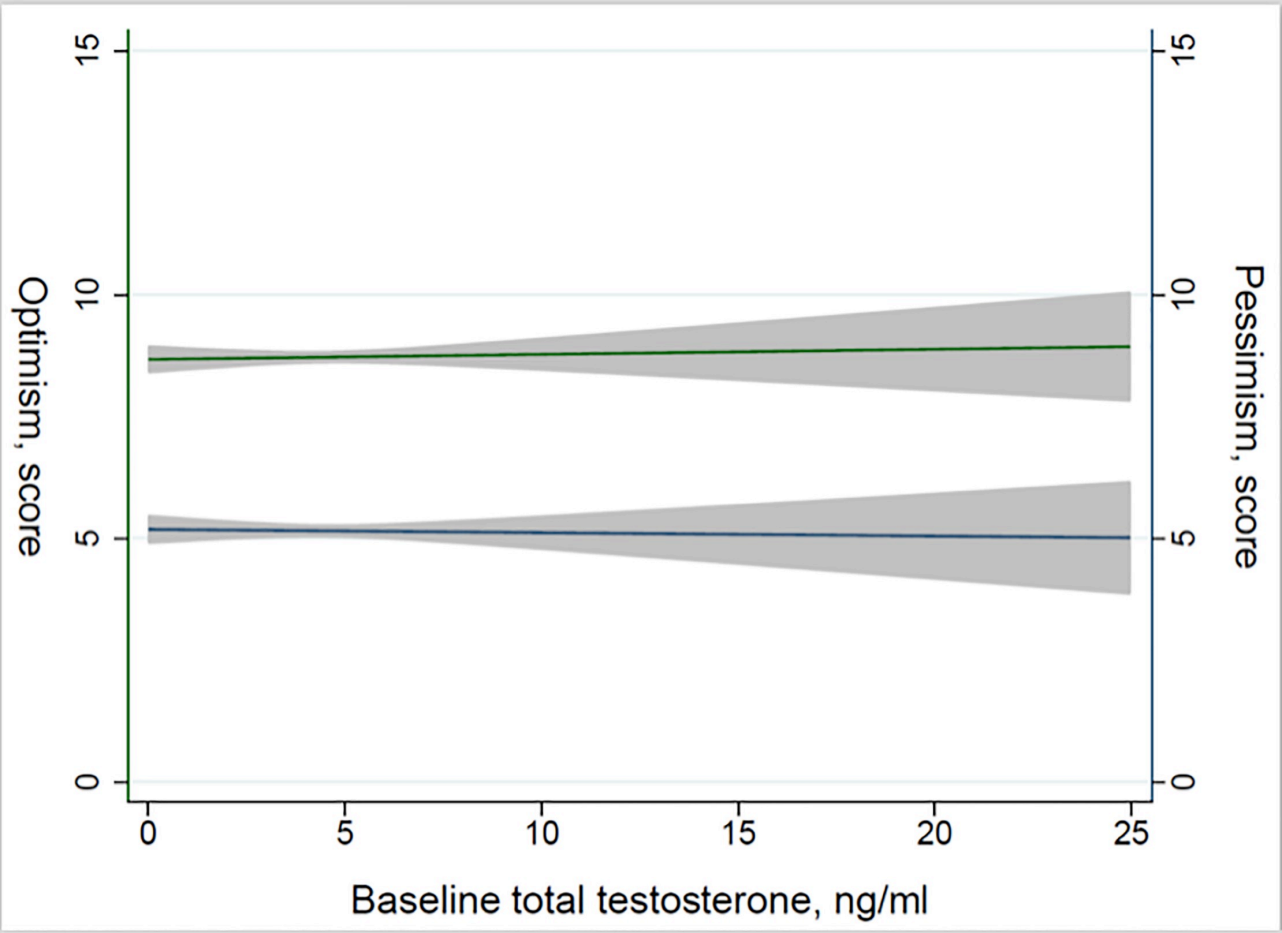
Optimism and pessimism by baseline testosterone among men. Linear regression for baseline optimism (green) and pessimism (blue) by baseline total testosterone concentrations. The green and blue lines represent the fitted regression line and the grey areas the 95% confidence intervals.

Sex-specific baseline characteristics of the study population are presented in Table 1. Cross-sectional analyses showed no association of T with optimism, pessimism or total LOT-R score among men and women (Table 2). Assessing a continuum of T concentrations and LOT-R scores, we observed no graded slope between T and optimism or pessimism (Fig 1). Longitudinal analyses of optimism, pessimism, and total LOT-R score yielded similarly non-significant results (multivariable-adjusted β-coefficients per one unit T change for LOT-R: men, -0.01 (95% CI: -0.24–0.22); women, 0.08 (-0.03–0.20)). Finally, one-year change in T was not related to optimism, pessimism or total LOT-R at four-year follow-up. None of the performed sensitivity analyses changed the revealed findings.

**Table 1:** Sex-specific baseline characteristics of the study population.

Variable	Women (N = 3,840)	Men (N = 2,653)	p-value*
**Age, years,**	56.8 (14.6)	58.4 (13.4)	< 0.01
**Total testosterone, ng/ml**	0.41 (0.27; 0.61)	4.4 (3.3; 5.5)	< 0.01
**One-year change in T, ng/ml**	-0.13 (-0.23; -0.04)	-0.07 (-0.73; 0.65)	< 0.01
**Waist circumference, cm**	90.1 (14.5)	101.6 (12.5)	< 0.01
**Current smoker, %**	19.9	22.9	< 0.01
**Physically inactive, %**	34.4	27.3	< 0.01
**Sobriety, %**	23.3	11.8	< 0.01
**Systolic blood pressure, mmHg**	130 (120, 140)	130 (120, 145)	< 0.01
**Diastolic blood pressure, mmHg**	80 (70; 85)	80 (75; 85)	< 0.01
**LOT-R, total score**			
**Baseline**	15 (13; 18)	15 (13; 18)	0.31
**1-year follow-up**	15 (13; 19)	16 (13; 18)	0.15
**4-year follow-up**	15 (13; 19)	16 (13; 19)	0.66
**LOT-R, optimism score**			
**Baseline**	9 (7; 11)	9 (7; 11)	0.28
**1-year follow-up**	9 (7; 11)	9 (7; 11)	0.19
**4-year follow-up**	9 (7; 11)	9 (7; 12)	0.07
**LOT-R, pessimism score**			
**Baseline**	5 (3; 7)	5 (3; 7)	0.21
**1-year follow-up**	5 (3; 7)	5 (3; 7)	0.07
**4-year follow-up**	5 (3; 7)	5 (3; 7)	0.40

Data are percentages, mean (SD) or median (Q1, Q3).

*Statistical comparisons were performed with χ 2 test (nominal data) or Mann-Whitney-U-test (continuous data). LOT-R, Revised Life-Orientation Test.

**Table 2:** Sex-specific associations of total testosterone with optimism, pessimism, and total LOT-R.

	Women	Men	Women	Men
	Cross-sectional		4-year follow-up	
**Optimism**	**β-coefficient**			
Baseline testosterone	0.12 (-0.01; 0.25)	0.008 (-0.05; 0.06)	0.04 (-0.11; 0.20)	0.02 (-0.05; 0.09)
Change in testosterone	-	-	-0.03 (-0.19; 0.13)	-0.07 (-0.18; 0.03)
**Pessimism**				
Baseline testosterone	0.01 (-0.12; 0.15)	0.005 (-0.05; 0.06)	0.05 (-0.08; 0.20)	-0.07 (-0.15; 0.004)
Change in testosterone	-	-	0.01 (-0.14; 0.18)	-0.02 (-0.13; 0.08)
**LOT-R, total score**				
Baseline testosterone	0.11 (-0.07; 0.29)	0.002 (-0.08; 0.08)	-0.01 (-0.24; 0.22)	0.08 (-0.03; 0.20)
Change in testosterone	-	-	-0.05 (-0.29; 0.19)	-0.04 (-0.20; 0.11)

Data are β-coefficients and their 95% confidence interval in multivariable models, respectively.

Multivariable models were adjusted for age, waist circumference, smoking status, physical inactivity, alcohol consumption, and blood pressure. Longitudinal analyses were additionally weighted for drop-out during follow-up.

LOT-R, Life Orientation Test revised.

## Discussion

To the best of our knowledge, this is the first observational study to investigate the association of T with optimism and pessimism in a large-scale sample of men and women. Taken together, cross-sectional and longitudinal analyses showed no association of T with dispositional optimism and pessimism, assessed by the LOT-R.

The tentative role of T as hormonal correlate of psychological traits stems from research in prenatal T exposure and economic decision-making. Using the right hand 2D:4D digit ratio (length of the index finger to the length of the ring finger) as putative marker for relative prenatal T exposure, the 2D:4D ratio has been shown to correlate with traits such as altruism [9], cooperation [10], and cognitive reflection [11]. Another line of research in economic decision-making suggests that T is linked to optimism and risk-taking [4], trust, and self-confidence [12]. But given their small scale, strongly selected samples, and narrow focus, the results of these studies are of low generalizability and do not allow for definite conclusions about a potential link between T and personality traits.

The absent association of endogenous T with dispositional optimism and pessimism in the present study provides a rationale for the lack of interventional evidence reporting no conclusive effect of exogenous T on personality, psychological well-being or mood in clinical trials [2]. Although men under T treatment occasionally show slightly better mood and lower severity of depressive symptoms, as reported by the long-awaited T-trials [13], the statistical significance of these findings does not compensate for their small effect size and unknown clinical significance. Also the suggested dose-response relationship between T and several domains of psychological well-being did not stand up to closer scrutiny. Exemplarily, a RCT among 44 healthy older men receiving five graded T doses over 20 weeks showed not effect on two measures of mood, including Hamilton's Depression Inventory and Young's Mania Scale [14].

Alternatively, change in T itself has been suggested as a relevant predictor of adverse health outcomes [15], risk taking behavior [16], and depressive symptoms [8]. However, on the level of psychological traits, the absence of an association of T change with optimism and pessimism in the present study is in line with synthesized evidence of a systematic review including 27 observational studies among healthy male adolescents, experiencing marked T changes during puberty, reporting no association of T change with behavior [17].

Although our findings agree with previous data showing low correlations between optimism and multiple laboratory parameters in the DETECT study, it is important to mention differences in the analyses of the LOT-R. While some studies investigated optimism and pessimism as two independent constructs [18], others assessed optimism as a continuous measure [19]. To address this methodological heterogeneity, we analyzed both: two-dimensional optimism and pessimism variables, as well as a continuous LOT-R score as one-dimensional trait. However, both analytical strategies yielded non-significant results.

The interpretation of the observational evidence presented here warrants consideration of the following strengths and potential limitations. Strengths of our study include the large sample size, repeated follow-up measures, and the comparative assessment of baseline T vs. T change in men as well as in women. Limitations may arise from the questionnaire-based assessment of LOT-R, relying on subjective and potentially inaccurate patient responses. Furthermore, our study is limited by the lack of data regarding additional sex hormones and alternative measures of the optimism-pessimism continuum, such as the Unrealistic Optimism Scale, the Hope Scale or the Optimism-Pessimism Scale [20].

In summary, the observational data reported here shows no association of T with optimism, pessimism or total LOT-R score, suggesting further research to elucidate potential direct and indirect associations between individual hormonal status and psychological traits.
